# A generalized additive model to disentangle age and diagnosis-specific cohort effects in psychological and behavioral outcomes in people living with HIV: the French cross-sectional ANRS-VESPA2 survey

**DOI:** 10.1186/s12889-019-6905-z

**Published:** 2019-05-17

**Authors:** Luis Sagaon-Teyssier, Antoine Vilotitch, Marion Mora, Gwenaëlle Maradan, Valérie Guagliardo, Marie Suzan-Monti, Rosemary Dray-Spira, Bruno Spire, VESPA2 study group, France Lert, Bruno Spire, Maria Patrizia Carrieri, Rosemary Dray-Spira, Christine Hamelin, Nicolas Lorente, Marie Préau, Marie Suzan-Monti, Fabienne Marcellin, Martin Duracinsky, Marion Mora, Gwenaëlle Maradan, Yann Le Strat, Lise Cuzin, Laurence Meyer, Daniela Rojas-Castro, Hugues Fischer, T. Allègre, P. Mours, J. M. Riou, M. Sordage, J. M. Chennebault, P. Fialaire, V. Rabier, M. Froidure, D. Huguet, D. Leduc, G. Pichancourt, A. Wajsbrot, C. Bourdeaux, A. Foltzer, B. Hoen, L. Hustache-Mathieu, S. Abgrall, R. Barruet, O. Bouchaud, A. Chabrol, S. Mattioni, F. Mechai, V. Jeantils, N. Bernard, F. Bonnet, M. Hessamfar, D. Lacoste, D. Malvy, P. Mercié, P. Morlat, F. Paccalin, M. C. Pertusa, T. Pistone, M. C. Receveur, M. A. Vandenhende, C. Dupont, A. Freire Maresca, J. Leporrier, E. Rouveix, S. Dargere, A. de la Blanchardière, A. Martin, V. Noyon, R. Verdon, O. Rogeaux, J. Beytout, F. Gourdon, H. Laurichesse, F. Meier, E. Mortier, A. M. Simonpoli, F. Cordier, I. Delacroix, V. Garrait, B. Elharrar, S. Dominguez, A. S. Lascaux, J. D. Lelièvre, Y. Levy, G. Melica, M. Buisson, L. Piroth, A. Waldner, N. Gruat, A. Leprêtre, P. de Truchis, D. Le Du, J. Cl. Melchior, R. Sehouane, D. Troisvallets, M. Blanc, I. Boccon-Gibod, A. Bosseray, J. P. Brion, F. Durand, P. Leclercq, F. Marion, P. Pavese, E. Brottier-Mancini, L. Faba, M. Roncato-Saberan, O. Bollengier-Stragier, J. L. Esnault, S. Leautez-Nainville, P. Perré, E. Froguel, M. Nguessan, P. Simon, P. Colardelle, J. Doll, C. Godin-Collet, S. Roussin-Bretagne, J. F. Delfraissy, M. Duracinsky, C. Goujard, D. Peretti, Y. Quertainmont, J. Marionneau, E. Aissi, N. Van Grunderbeeck, E. Denes, S. Ducroix-Roubertou, C. Genet, P. Weinbreck, C. Augustin-Normand, A. Boibieux, L. Cotte, T. Ferry, J. Koffi, P. Miailhes, T. Perpoint, D. Peyramond, I. Schlienger, J. M. Brunel, E. Carbonnel, P. Chiarello, J. M. Livrozet, D. Makhloufi, C. Dhiver, H. Husson, A. Madrid, I. Ravaux, M. L. de Severac, M. Thierry Mieg, C. Tomei, S. Hakoun, J. Moreau, S. Mokhtari, M. J. Soavi, O. Faucher, A. Ménard, M. Orticoni, I. Poizot-Martin, M. J. Soavi, N. Atoui, V. Baillat, V. Faucherre, C. Favier, J. M. Jacquet, V. Le Moing, A. Makinson, R. Mansouri, C. Merle, N. Elforzli, C. Allavena, O. Aubry, M. Besnier, E. Billaud, B. Bonnet, S. Bouchez, D. Boutoille, C. Brunet, N. Feuillebois, M. Lefebvre, P. Morineau-Le Houssine, O. Mounoury, P. Point, F. Raffi, V. Reliquet, J. P. Talarmin, C. Ceppi, E. Cua, P. Dellamonica, F. De Salvador-Guillouet, J. Durant, S. Ferrando, V. Mondain-Miton, I. Perbost, S. Pillet, B. Prouvost-Keller, C. Pradier, P. Pugliese, V. Rahelinirina, P. M. Roger, E. Rosenthal, F. Sanderson, L. Hocqueloux, M. Niang, T. Prazuck, P. Arsac, M. F. Barrault-Anstett, M. Ahouanto, E. Bouvet, G. Castanedo, C. Charlois-Ou, A. Dia Kotuba, Z. Eid-Antoun, C. Jestin, K. Jidar, V. Joly, M. A. Khuong-Josses, N. Landgraf, R. Landman, S. Lariven, A. Leprêtre, F. L’hériteau, M. Machado, S. Matheron, F. Michard, G. Morau, G. Pahlavan, B. C. Phung, M. H. Prévot, C. Rioux, P. Yéni, F. Bani-Sadr, A. Calboreanu, E. Chakvetadze, D. Salmon, B. Silbermann, D. Batisse, M. Beumont, M. Buisson, P. Castiel, J. Derouineau, M. Eliaszewicz, G. Gonzalez, D. Jayle, M. Karmochkine, P. Kousignian, J. Pavie, I. Pierre, L. Weiss, E. Badsi, M. Bendenoun, J. Cervoni, M. Diemer, A. Durel, A. Rami, P. Sellier, H. Ait-Mohand, N. Amirat, M. Bonmarchand, F. Bourdillon, G. Breton, F. Caby, J. P. Grivois, C. Katlama, M. Kirstetter, L. Paris, F. Pichon, L. Roudière, L. Schneider, M. C. Samba, S. Seang, A. Simon, H. Stitou, R. Tubiana, M. A. Valantin, D. Bollens, J. Bottero, E. Bui, P. Campa, L. Fonquernie, S. Fournier, P. M. Girard, A. Goetschel, H. F. Guyon, K. Lacombe, F. Lallemand, B. Lefebvre, J. L. Maynard, M. C. Meyohas, Z. Ouazene, J. Pacanowski, O. Picard, G. Raguin, P. Roussard, M. Tourneur, J. Tredup, N. Valin, S. Balkan, F. Clavel, N. Colin de Verdière, N. De Castro, V. de Lastours, S. Ferret, S. Gallien, V. Garrait, L. Gérard, J. Goguel, M. Lafaurie, C. Lascoux-Combe, J. M. Molina, E. Oksenhendler, J. Pavie, C. Pintado, D. Ponscarme, W. Rozenbaum, A. Scemla, P. Bonnard, L. Lassel, M. G. Lebrette, T. Lyavanc, P. Mariot, R. Missonnier, M. Ohayon, G. Pialoux, M. P. Treilhou, J. P. Vincensini, J. Gilquin, B. Hadacek, L. Nait-Ighil, T. H. Nguyen, C. Pintado, A. Sobel, J. P. Viard, O. Zak Dit Zbar, H. Aumaître, A. Eden, M. Ferreyra, F. Lopez, M. Medus, S. Neuville, M. Saada, L. Blum, P. Perfezou, C. Arvieux, J. M. Chapplain, M. Revest, F. Souala, P. Tattevin, S. Bord, F. Borsa-Lebas, F. Caron, C. Chapuzet, Y. Debab, I. Gueit, M. Etienne, C. Fartoukh, K. Feltgen, C. Joly, S. Robaday-Voisin, P. Suel, M. A. Khuong, J. Krausse, M. Poupard, G. Tran Van, C. Cazorla, F. Daoud, P. Fascia, A. Frésard, C. Guglielminotti, F. Lucht, C. Bernard-Henry, C. Cheneau, J. M. Lang, E. de Mautort, M. Partisani, M. Priester, D. Rey, C. Majerholc, D. Zucman, A. Assi, A. Lafeuillade, J. P. de Jaureguiberry, O. Gisserot, C. Aquilina, F. Prevoteau du Clary, M. Alvarez, M. Chauveau, L. Cuzin, P. Delobel, D. Garipuy, E. Labau, B. Marchou, P. Massip, M. Mularczyk, M. Obadia, F. Ajana, C. Allienne, V. Baclet, X. de la Tribonnière, T. Huleux, H. Melliez, A. Meybeck, B. Riff, M. Valette, N. Viget, F. Bastides, L. Bernard, G. Gras, P. Guadagnin, T. May, C. Rabaud, A. Dos Santos, Y. Poinsignon, O. Derradji, L. Escaut, E. Teicher, D. Vittecoq, J. Bantsima, P. Caraux-Paz, O. Patey

**Affiliations:** 1 Aix Marseille Univ, INSERM, IRD, SESSTIM, Sciences Economiques & Sociales de la Santé & Traitement de l’Information Médicale, 27 Bd Jean Moulin, 13385 Marseille Cedex 5, France; 2 ORS PACA, Southeastern Health Regional Observatory, 27 Bd Jean Moulin, 13385 Marseille Cedex 5, France; 3 INSERM, UMR_S1136, Pierre Louis Institute of Epidemiology and Public Health, Team Research in Social Epidemiology, 56, Boulevard Vincent Auriol - CS 81393 – 75646, F-75013 Paris Cedex 13, France; 4 Pierre Louis Institute of Epidemiology and Public Health, Team Research in social epidemiology, Sorbonne Universités, UPMC Univ Paris 06, UMR_S1136, 56, Boulevard Vincent Auriol - CS 81393 – 75646, F-75013 Paris Cedex 13, France

**Keywords:** Age, Cohort effects, Quality of life, Material and moral support, HIV-status disclosure, Semi-parametric GAM

## Abstract

**Background:**

Unlike their younger counterparts, some of today’s older HIV patients were diagnosed before the advent of highly active antiretroviral therapy (HAART). The psychosocial and behavioral outcomes of people living with HIV (PLWH) have been widely studied, and associated factors are well known. However, their evolution both in terms of age and diagnosis-specific cohort effects is not well understood.

**Methods:**

Data from the ANRS-VESPA2 cross-sectional survey, representative of French PLWH, were used to investigate whether psychosocial and behavioral outcomes such as quality of life, need for support and HIV status disclosure, evolve under both the influence of patients’ age and diagnosis-specific cohort effects. A semi-parametric generalized additive model (GAM) was employed. The physical and mental components of health-related quality of life, the need for material and moral support, and HIV-status disclosure, constituted our outcomes.

**Results:**

Non-linear diagnosis-specific cohort effects were found for physical and mental QoL and HIV-status disclosure. Overall, physical QoL was better in recently diagnosed patients than in those diagnosed in the early 1980s. An increasing influence of diagnosis-specific cohort effects between 1983 and 1995 was observed. No cohort effects were noticeable between 1996 and 2000, while an increasing influence was apparent for patients diagnosed with HIV from 2000 to 2011 (year of study). For mental QoL, the only increase was observed in participants diagnosed with HIV between 1983 and 2000. The relationship between diagnosis-specific cohort effects and HIV status disclosure was negative overall: participants diagnosed after 2000 were much less likely to disclose than those diagnosed before 1995. The effect of age was significantly associated with all outcomes, with a non-linear influence on mental QoL and with the need for material/moral support.

**Conclusions:**

Psychosocial and behavioral outcomes are complex processes which can be explained in different ways by a combination of the clinical and social contexts which PLWH are exposed to at the time of diagnosis, and by developmental characteristics. A greater understanding of these processes could inform healthcare policy-making for specific HIV generations and different HIV age groups.

## Background

The psychosocial and behavioral outcomes of people living with HIV (PLWH) have been widely studied, and associated factors are well known. However, no study to date has simultaneously investigated the influence which both age and human immunodeficiency virus (HIV) diagnosis-related cohort effects may have on these outcomes, specifically because of the methodological difficulties arising from their linear dependency. Disentangling these two influences from one another is important in order to understand whether psychosocial and behavioral outcomes of PLWH are processes driven by individual-related characteristics such as age, and/or by contextual-related aspects. However, this is methodologically challenging for several reasons: i) the complex composition of the population of PLWH in terms of age and year of diagnosis, ii) the complex evolution of HIV care, which has seen improvements in diagnosis, treatment and health care programs lead to HIV becoming a chronic disease, and iii) the relationship between the timing of these improvements and aging. More specifically, unlike their younger HIV counterparts, some of today’s older patients were diagnosed and treated before the advent of highly active antiretroviral therapy (HAART) with serious negative consequences on their health-related quality of life due to the toxicity of older drugs such as stavudine, zidovudine and nevirapine [1]. In addition, aging with HIV is not only related to clinical aspects such as antiretroviral-related side effects, comorbidities and pain, but also to stigma [2, 3]. Furthermore, mental and physical health may be affected by the isolation of PLWH and the consequent difficulties they encounter with regard to HIV disclosure, especially for older people [4, 5]. In this study, we argue that variations in psychosocial and behavioral outcomes among PLWH can be attributed not only to individual differences but also to the different clinical and social contexts which they were confronted with at the time of HIV diagnosis, represented by diagnosis-specific cohort effects.

While studies on psychosocial and behavioral outcomes among PLWH often focus on the relationship between age and outcomes, to date the question of diagnosis-specific cohort effects has only been studied indirectly, by specifying a variable accounting for the time elapsed since HIV diagnosis. No study has ever simultaneously explored the influence of both of these factors in the same model. In terms of patient’s age, existing literature has suggested a modest or null effect on quality of life (irrespective of the instrument used HAT-HRQL, WHOQOL, SF-36 or SF-12) [6–10]. However, contrasting results have been found regarding its effect on disclosure, with one study indicating a relatively higher probability of disclosure in older PLWH [11], while another reported a significant effect in bivariate analysis which was no longer significant in multivariate analysis [12]. In studies using a more detailed definition of disclosure – one where the degree of disclosure was specified (i.e., broad, selective or no disclosure) [13, 14], and another where the persons to whom participants disclosed were specified (i.e., partner, family, friend, colleague, etc.) [9] – no effect of age on disclosure was observed. In terms of diagnosis-specific cohort effects on disclosure, no study to date has yet addressed this relationship, although there is evidence suggesting that the time elapsed since HIV diagnosis is positively related to disclosure [15], implying that patients in newer cohorts are less likely to disclose their HIV status.

In France, quality of life (QoL) analyses in the cross-sectional study ANRS-VESPA1 (2003) noted that older PLWH were more likely to report an acceptable physical quality of life (PQoL), whereas no significant age effect was found for mental quality of life (MQoL). However, when specifically distinguishing between PLWH diagnosed before/after 1996 (i.e., reflecting the pre-HAART and HAART eras), the results from the same data revealed no significant effect either for PQoL or MQoL [16]. Analyses of data from the ANRS-VESPA2 cross-sectional study (2011) showed that age was negatively associated with PQoL but positively associated with MQoL. However, diagnosis-specific cohort effects were not analyzed with these data, and only a negative association was found between time since HIV diagnosis and both QoL dimensions [17].

One of the difficulties of simultaneously studying age and diagnosis-specific cohort effects is linked to the strong potential linear dependency between these two explanatory factors. Although some studies attempt to address this issue [18–22], most (including those on HIV) do it only superficially. Indeed, authors often circumvent this linear dependency either by aggregating or stratifying age and/or cohort in categories, resulting in the waste of important information [23, 24]. The objective of the present paper was to implement a tailored methodological framework to assess the extent to which the patterns of QoL, of material/moral support needs, and of HIV-status disclosure are related to diagnosis-specific cohort effects, to age, and to a combination of both these factors.

## Methods

### Study design and participants

Data were used from the ANRS-VESPA2 survey (ANRS is the French National Agency of research on AIDS and viral hepatitis; VESPA2 is the acronym for “VIH: Enquête sur les Personnes Atteintes”), which is representative of PLWH followed up in 2011 in 73 French care units with caseloads of more than 100 HIV+ patients. Eligibility criteria were > 18 years old, diagnosed as HIV+ and living in France for at least 6 months. The 3022 patients included in the sample provided information about their living conditions including socio-demographic, economic, psychosocial and behavioral aspects, in a face-to-face interview. A self-administered questionnaire collecting other patient information was used to assess QoL, self-perception of health status, and quality of HIV care received. HIV care staff provided medical patient-related information about key indicators of HIV, comorbidities, treatments and hospital-related characteristics (see Dray-Spira et al. [25]). The survey was approved by the French ethical committee (CCTIRS, 20/01/2010) and the Commission for data protection (CNIL, Decision DR-2010-368, 06/12/2010).

### Psychosocial and behavioral outcomes

The PQoL and MQoL scores for health related quality of life (HRQL) were calculated using the SF-12 scale included in the self-administered questionnaire [26]. These scores are continuous variables ranging from 0 to 100, with higher scores denoting better HRQL. To assess respondents’ need for material/moral support, two dichotomous variables were constructed as follows: 1 = support needed in the previous 12 months (whether received or not), and 0 = no support needed. Finally, a dichotomous variable indicated whether a patient had disclosed (=1) or not (=0) his/her HIV-status to at least one relative or close acquaintance.

### Statistical analysis

Descriptive statistics were performed for the outcomes considered in this study, and potential explanatory factors were selected from bivariate analysis using Fisher’s test and median tests for categorical and continuous variables, respectively. A semi-parametric generalized additive model (GAM) was implemented for each outcome with the following form:

1 $$ \mathrm{g}\left(\mathrm{mean}\ \mathrm{outcome}\right)=\upalpha +\upbeta \mathrm{X}+{\mathrm{f}}_1\left(\mathrm{cohort}\right)+{\mathrm{f}}_2\left(\mathrm{age}\right) $$

In this expression, α + βX is the parametric portion of the model where α is the intercept and β is the vector of parameters associated with the set of explanatory variables X. The non-parametric part of expression (1) is formed by f_1_(.) and f_2_(.), which are smooth functions for diagnosis-specific cohort and age effects, respectively. These two functions describe the relationship between these explanatory factors and the studied outcomes. The functions are associated with estimated degrees of freedom (EDF) that indicate whether the relationship is linear (EDF = 1) or non-linear (EDF > 1). One of the main advantages of the GAM implemented in this study is the possibility to address the issue of linear dependency (i.e. collinearity) [27] between diagnosis-specific cohort and age effects which often impedes their simultaneous estimation. Estimations for the two factors’ effects on PQoL and MQoL scores were carried out by specifying a linear structure (i.e., the g(.) was the identity link function), whereas for the need for material/moral support and HIV disclosure, a probit function was used (i.e., g(.) was the probit link function). Sociodemographic, socioeconomic and/or clinical-related patient characteristics were controlled for in the parametric portion of the model. R software was used to perform all the statistical analyses in this article [28], and the “mgcv” package for GAM estimation [29].

## Results

PQoL and MQoL scores were computed for the 2267 (out of 3022) participants who answered all 12 items of the SF-12 scale measuring HRQL (i.e., a response rate of 75%) included in the self-administered questionnaire. Median scores for PQoL and MQoL were 48.4 IQR [40.6–54.6] and 45.1 IQR [33.7–53.2], respectively. With regard to the need for material and moral support, of the 3006 participants for whom information was available, 55.9 and 80.6%, respectively, declared they needed support. Finally, 3016 participants declared they had disclosed their HIV status to at least one relative or close acquaintance.

### Parametric portion of the model

Parametric estimates are presented in Table 1. PQoL scores were lower for women (coeff = − 1.3, 95% CI [− 2.2;-0.5]), for Sub-Saharan African immigrants (coeff = − 2.6, 95% CI [− 3.7;-1.5]), for participants perceiving both difficult financial situations (coeff = − 2.5, 95% CI [− 3.3;-1.6]) and care-related discrimination (coeff = − 3.1, 95% CI [− 4.3;-1.9]), for patients who had experienced AIDS-defining malignancies (coeff = − 1.8, 95% CI [− 2.6;-1.0]) and for HCV co-infected patients (coeff = − 1.7, 95% CI [− 2.7;-0.7]). On the contrary, employed patients (coeff = 2.9, 95% CI [2.1;3.6]) and those with a high level of education (coeff = 1.3, 95% CI [0.1;2.4]) had higher PQoL scores. Higher scores for MQoL were found for Sub-Saharan African immigrants (coeff = 5.0, 95% CI [3.6;6.4]), married and cohabitating participants (coeff = 1.9, 95% CI [1.0;2.9]), participants with a positive perception of their financial situation (coeff = 1.7, 95% CI [0.5;3.0]), and those who had never experienced AIDS-defining malignancies (coeff = 1.6, 95% CI [0.6,2.7]).

**Table 1:** Semi-parametric generalized additive model (GAM) for psychosocial and behavioral outcomes among PLWH in France (ANRS-VESPA2 survey)

	Physical QoL	Mental QoL	Need for material support	Need for moral support	HIV-status disclosure *N* = 3016
	*N* = 2267		*N* = 3006		*N* = 3016
Parametric part of the model					
	Coeff. [95% CI]	Coeff. [95% CI]	Coeff. [95% CI]	Coeff. [95% CI]	Coeff. [95% CI]
Intercept	50.6 [49.4;51.7]^c^	42.0 [41.0;43.1]^c^	0.0 [−0.2;0.2]	0.8 [0.6;1.1]^c^	2.3 [1.8;2.7]^c^
Sex					
Male	ref	ref	ref	ref	ref
Female	−1.3 [− 2.2;-0.5]^b^	− 1.0 [− 2.1;0.1]	0.4 [0.2;0.6]^c^	1.1 [0.8;1.4]^c^	0.6 [0.2;1.1]^b^
MSM					
Yes				0.4 [0.2;0.7]^c^	0.5 [0.0;1.0]^a^
No				ref	ref
Immigrant from Sub-Saharan Africa					
Yes	−2.6 [−3.7;-1.5]^c^	5.0 [3.6;6.4]^c^	0.8 [0.5;1.1]^c^		−1.1 [−1.5;−0.6]^c^
No	ref	ref	ref		ref
Marital status					
Married or cohabitating		1.9 [1.0;2.9]^c^	-0.6 [−0.8;-0.4]^c^		1.4 [0.9;1.8]^c^
Single, divorced or widowed		ref	ref		ref
Children					
Yes			0.2 [0.0;0.4]^a^		
No			ref		
Professional status					
Employed	2.9 [2.1;3.6]^c^	2.1 [1.1;3.1]^c^	−0.5 [−0.7;-0.3]^c^		
Other (unemployed, inactive)	ref	ref	ref		
Education level					
< High school	ref				ref
= High school	−0.4 [−1.5;0.6]				0.6 [0.2;1.0]^b^
> High school	1.3 [0.1;2.4]^a^				0.5 [0.0;0.9]
Perceived care-related discrimination (in the previous 2 years)					
Yes	−3.1 [− 4.3;-1.9]^c^	− 4.2 [− 5.7;2.6]^c^	0.5 [0.2;0.8]^b^	0.4 [0.0;0.8]^a^	
No	ref	ref	ref	ref	
Perceived financial situation					
Difficult	−2.5 [−3.3;-1.6]^c^	−4.9 [− 6.0;-3.8]^c^	1.7 [1.4;1.9]^c^	0.7 [0.5;1.0]^c^	
Adequate	ref	ref	ref	ref	
Comfortable	0.8 [−0.2;1.9]	1.7 [0.5;3.0]^b^	−1.1 [−1.3;-0.8]^c^	− 0.2 [− 0.5;0.0]^a^	
Beneficiary of free universal health coverage					
Yes			0.8 [0.4;1.1]^c^		
No			ref		
Experienced AIDS-related malignancies					
Yes	−1.8 [−2.6;-1.0]^c^	1.6 [0.6;2.7]^b^			
No	ref	ref			
HCV Co-infection					
Yes	−1.7 [−2.7;-0.7]^c^			0.6 [0.3;0.9]^c^	
No	ref			ref	
Non-parametric portion of the model					
	EDF	EDF	EDF	EDF	EDF
Cohort (year of diagnosis)	3.2^b^	2.2^c^	1.7	2.1	3.7^c^
Age	1.1^c^	6.2^c^	7.7^c^	8.6^c^	1.4^c^

Significance confidence level: ^a^10%,^b^5% and ^c^1%

Probit estimates show that need for material support was more likely in women (coeff = 0.4, 95% CI [0.2;0.6]), Sub-Saharan African immigrants (coeff = 0.8, 95% CI [0.5;1.1]), and participants with children (coeff = 0.2 95% CI [0.0;0.4]). Participants reporting unfavorable socioeconomic characteristics were more likely to need material support, specifically those who declared a difficult financial situation (coeff = 1.7, 95% CI [1.4;1.9]) and beneficiaries of free universal health coverage (coeff = 0.8, 95% CI [0.4;1.1]), which is allocated only to people on low income and those unemployed in France. On the contrary, married (coeff = − 0.6, 95% CI [− 0.8;-0.4]) and employed (coeff = − 0.5, 95% CI [− 0.7;-0.3]) participants were less likely to need material support. Participants’ perception of care-related discrimination was associated with an increased probability of needing material support (coeff = 0.5, 95% CI [0.2;0.8]), whereas no health-related factors were associated with this outcome. Women (coeff = 1.1, 95% CI [0.8;1.4]) and those with difficult financial situations (coeff = 0.7, 95% CI [0.5;1.0]) were more likely to need moral support. The probability of needing moral support was also higher in MSM (coeff = 0.4, 95% CI [0.2;0.7]) and HCV co-infected patients (coeff = 0.6, 95% CI [0.3;0.9]).

The last column in Table 1 shows the probit estimation for HIV-status disclosure. This outcome was associated with sociodemographic characteristics. Women (coeff = 0.6, 95% CI [0.2;1.1]), married participants (coeff = 1.4, 95% CI [0.9;1.8]) were more likely to disclose their HIV status. MSM also tended to be more likely to disclose. Finally, Sub-Saharan African immigrants (coeff = − 1.1, 95% CI [− 1.5;-0.6]) were less likely to disclose.

Fixed-effects for hospitals were specified for all models in order to control for hospital-related heterogeneity in our outcomes (results not-shown). However, heterogeneity appeared to be individual-related rather than hospital-related as fixed-effects were not significant.

### Non-parametric portion of the model: diagnosis-specific cohort effects and age effect

**Fig. 1:**
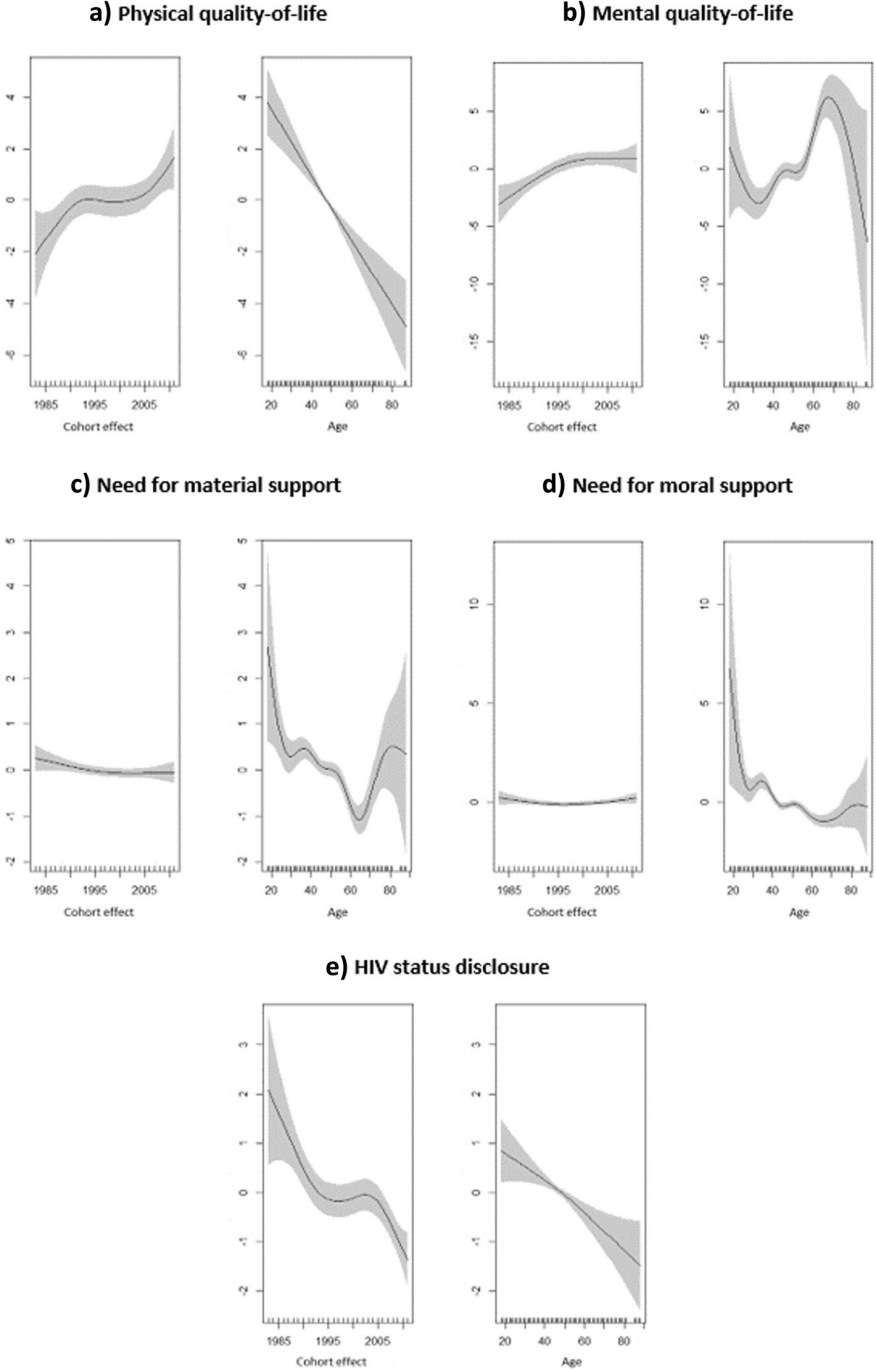
Non-parametric estimation of diagnosis-specific cohort effects and the effect of age

The effect of age and of diagnosis-specific cohort effects were assessed non-parametrically over the five outcomes analyzed in this article and are presented at the bottom of Table 1. The GAM estimation indicated, through the significant EDF associated with diagnosis-specific cohort effects, that the time at which participants were diagnosed with HIV influenced MQoL (EDF = 2.2, *p* < 0.01), PQoL (EDF = 3.2, *p* < 0.05) and HIV-status disclosure (EDF = 3.7, *p* < 0.01). More specifically, diagnosis-specific cohort effects were nonlinear, with large fluctuations over time being observed, principally for the latter two outcomes as shown in Fig. 1a and e, respectively. Overall, PQoL was better in patients recently diagnosed than in those diagnosed in the early 1980s. An increasing influence of diagnosis-specific cohort effects between 1983 and 1995 was observed. No cohort effects were noticeable between 1996 and 2000, while an increasing influence was apparent for patients diagnosed with HIV after 2000. The influence of diagnosis-specific cohort effects on MQoL fluctuated less (Fig. 1b), with the only increase being observed in participants diagnosed with HIV between 1983 and 2000. Patients diagnosed after this period did not differ in terms of MQoL. Finally, the relationship between diagnosis-specific cohort effects and HIV status disclosure was negative overall: participants diagnosed after 2000 were much less likely to disclose than those diagnosed before 1995. Between 1995 and 2000 no diagnosis-specific cohort effect on HIV status disclosure was observed (Fig. 1e).

The effect of age was significant for the 5 outcomes analyzed. It was linear for both PQoL (EDF = 1.1, *p* < 0.01) (see Fig. 1a) and HIV status disclosure (EDF = 1.4, *p* < 0.01), but non-linear with large fluctuations for MQoL (EDF = 6.2, *p* < 0.01) (see Fig. 1b), the need for material support (EDF = 7.1, *p* < 0.01) and for moral support (EDF = 8.6, *p* < 0.01). With respect to MQoL, Fig. 1b shows a decrease for those between 18 and 30 years old, followed by an increase for those between 31 and 64 years old, and another decrease for those 65 and older. The effect of age on the need for material support and for moral support was similar, although more pronounced for the former (Fig. 1c and d). More specifically the probability of needing material or moral support decreased in those between 20 and 65 years old, then increased in those up to 80 years of age before it started to decrease again. Finally, a negative linear relationship between age and the probability of disclosing HIV status was found, with older participants being more likely to disclose than younger patients (Fig. 1e).

## Discussion

Our results suggest that PQoL is primarily driven by diagnosis-specific cohort effects, which may be attributable to exposure to different clinical contexts over time. In contrast, the evolution of MQoL appears to be an individual process which changes with age. HIV status disclosure was driven by the combined effect of age at the time of diagnosis and the year of diagnosis. Our semi-parametric generalized additive model on data from the ANRS-VESPA2 study confirmed the importance and value of disentangling diagnosis-specific cohort effects from age effects in order to understand the interplay between contextual and developmental influences on the evolution of psychosocial and behavioral outcomes among PLWH. The particular evolution of the need for moral and material support shows that these outcomes are individual processes that differ from one another only in terms of age. In contrast, with regard to QoL, the PQoL and MQol processes appear to be more complex and are the result of the combination of both diagnostic-specific cohort effects (for PQoL) and the effect of age (MQoL).

Improved survival rates, thanks to the efficiency of HAART, have led to aging with HIV becoming an important research subject, from both biological and clinical points of view. [30]. Indeed some processes are accelerated (e.g., in the immunological system), whereas others (e.g., organ-specific processes) are more accentuated than accelerated, leading to morbidity [31]. The literature also notes that PLWH are more likely to experience psychiatric problems and have relatively higher suicide tendencies, especially those aged 65 and over [32]. However, much less is known about the association between aging and psychosocial/behavioral outcomes in PLWH, and the evolution of these outcomes in terms of the social and care contexts (with diagnostic-specific cohort effects being proxies) at the time of diagnosis.

The positive diagnostic-specific cohort effects found for PQoL and MQoL are in line with existing literature and may be linked to improvements in treatment efficacy and tolerability as well as increased effectiveness of tailored HIV care programs that reflect the different care contexts PLWH experienced depending on the year in which they were diagnosed [33]. New therapeutic HIV regimens have been associated with better quality of life through improved physical health [34–36]. Nevertheless, this positive diagnosis-specific cohort effect is countered by the fact that PQoL decreases as people get older, an effect that is related either to comorbidities specific to HIV [37, 38] or to aging [39]. The modest diagnosis-specific cohort effects estimated for MQoL contrast with the fluctuating pattern seen for the effect of age.

The present study was not made without limitations. The first, concerned the naturally upward bias of the outcomes in relation with the original cohorts of diagnosed individuals. This limitation is often found in studies about health-related outcomes as analyses are carried out on data observed among survivors. Indeed, PLWH who died after diagnosis and before the beginning of the survey in 2011 were, unsurprisingly, less healthy than those included in the survey. A second limitation of this study concerns the unrepresentativeness of the ANRS-VESPA2 with respect to the whole population of PLWH in France. Indeed, participants were enrolled directly enrolled in HIV and exclusion criteria were: being unaware of the HIV-positive status; being diagnosed < 6 months before the survey; being diagnosed > 6 months before the survey but not receiving medical care for HIV infection; or being diagnosed > 6 months before the survey but treated either in HIV care units with caseloads under 100 patients or followed up by general practitioners outside hospital care units [25].

To our knowledge, this is the first time that the effects of age and of diagnosis-specific cohort effects on psychosocial and behavioral outcomes of PLWH have been studied simultaneously as explanatory variables. Our results show that the evolution of psychosocial and behavioral outcomes is a complex process which depends on the context which PLWH are exposed to at the time of diagnosis, by developmental characteristics as suggested by the effect of age, and by a combination of both.

## Conclusions

The evolution of PLWH outcomes cannot be completely explained by aging. The clinical and social contexts at the time of HIV diagnosis also play a role. Understanding this evolution may lead to more tailored HIV healthcare policies which take into account different HIV generations and/or different age groups, depending on which psychosocial and behavioral aspects of PLWH need to be improved.

## Abbreviations

EDF: Estimated degrees of freedom
GAM: Generalized additive model
HAART: Highly active antiretroviral therapy
HIV: Human immunodeficiency virus
HRQL: Health-related quality of life
MQoL: Mental quality of life
PLWH: People living with HIV
PQoL: Physical quality of life
QoL: Quality of life
VESPA2: “VIH: Enquête sur les Personnes Atteintes”
